# Neonatal sepsis in a tertiary health facility in Cape Coast, Ghana

**DOI:** 10.1371/journal.pone.0302533

**Published:** 2024-05-08

**Authors:** Joshua Panyin Craymah, Derek Anamaale Tuoyire, Portia Adjei-Ofori, Oluwayemisi Esther Ekor, Paul Aduoku Ninson, Milton Henschel Kojo Armoh Ewusi

**Affiliations:** 1 Department of Internal Medicine and Therapeutics, School of Medical Sciences, University of Cape Coast, Cape Coast, Ghana; 2 Department of Community Medicine, School of Medical Sciences, University of Cape Coast, Cape Coast, Ghana; 3 Department of Pediatrics, Cape Coast Teaching Hospital, Cape Coast, Ghana; 4 Department of Anaesthesia and Pain Management, School of Medical Sciences, University of Cape Coast, Cape Coast, Ghana; 5 Department of Biostatistics, Cape Coast Teaching Hospital, Cape Coast, Ghana; Arba Minch University, ETHIOPIA

## Abstract

**Background:**

Neonatal Sepsis remains a significant burden globally, accounting for over 2.5 million neonatal deaths annually, with low-and middle-income countries (LMIC) including Ghana disproportionately affected. The current study sought to ascertain the prevalence of neonatal sepsis and associated factors based on analysis of institutional records from Cape Coast Teaching Hospital (CCTH) in Ghana.

**Methods:**

The study involved a retrospective cross-sectional review of randomly sampled medical records of 360 neonates CCTH from January 2018 to December 2021. Descriptive proportions and binary logistic regression analysis were conducted to estimate the prevalence of neonates with sepsis and associated factors.

**Results:**

The prevalence of neonates with sepsis over the period was estimated to be 59%, with early-onset neonatal sepsis (EONS) and late-onset neonatal sepsis (LONS) accounting for about 29% and 30%, respectively. Neonatal factors associated with sepsis were low Apgar score (AOR = 1.64; 95% CI:1.01–2.67, p = 0.047) and low birth weight (AOR = 2.54; 95% CI:1.06–6.09, p = 0.037), while maternal factors were maternal education (AOR = 2.65; 95% CI:1.04–6.7, p = 0.040), caesarean deliveries (AOR = 0.45; 95% CI:0.26–0.75, p = 0.003), maternal infection (AOR = 1.79; 95% CI:1.09–2.94, p = 0.020) and foul-smelling liquor (AOR = 1.84; 95% CI:1.09–3.07, p = 0.020).

**Conclusion:**

The study underscores the need for improved routine care and assessment of newborns to prevent the onset of neonatal sepsis, with particular emphasis on the neonatal and maternal risk factors highlighted in the current study.

## Introduction

The critical nature of the neonatal period (first 28 days of life) for child survival cannot be overemphasized. An estimated 2.5 million infants die within their first month of life annually, representing about half of deaths in children under 5 years of age [1]. Neonatal sepsis accounts for a significant proportion of all infection-related mortality and morbidity among infants within the neonatal period. Although the global burden of neonatal sepsis is difficult to ascertain, modelled data from the United Nations Inter-Agency Group for Child Mortality Estimation (UN IGME) suggest that 375,000 neonatal deaths resulted from sepsis across the globe in 2018 [2]. Estimates based on systematic review and meta-analyses of studies between 1979 and 2019 report the worldwide number of neonatal sepsis cases to be between 1.3 to 3.9 million annually, with deaths ranging from 400,000 to 700,000 [3].

The available evidence on the burden of neonatal sepsis point to significant disparities with low- and middle-income countries (LMICs) disproportionately affected. According to the Global Sepsis Alliance, the rate neonatal sepsis is about 40 times higher in LMICs while deaths are twice as high compared with more advanced countries [4]. The picture in sub-Saharan Africa is no different with about 35 neonatal deaths occurring per 1,000 live births [5].

Previous studies have documented a number of risk factors for neonatal sepsis which could generally be classified as maternal, neonatal and hospital care related factors [6–8]. Maternal factors typically involve factors which result in the transmission of infections from mother to foetus or neonate including urinary tract infection, premature rupture of membranes (PROM), chorioamnionitis, early breast feeding, place of delivery, prolonged labour cord care, mode of delivery and maternal demographics such as age [9]. Factors at the level of the neonate often relate to immunological immaturity which increases their susceptibility to infections. These include prematurity, sex, rashes, congenital abnormality, low Apgar and neonatal resuscitation [10]. Hospital related factors are generally associated with nosocomial infections from prolonged hospital admission, poor hygiene, invasive procedures, superficial infection, non-lacteal feeding among others [11, 12].

Although prior studies on neonatal sepsis and associated factors in LMICs abound, there is limited literature on the subject in Ghana. The few prior studies [13–15] in Ghana have mainly been conducted within the context of the nation’s capital, Accra, with little insights from other regions. With the view to extending the discourse neonatal sepsis in Ghana, the current study sought to ascertain the prevalence of neonatal sepsis and associated factors based on analysis of institutional records from Cape Coast Teaching hospital (CCTH) in Ghana. Considering that CCTH serves as a referral tertiary facility for both the Central and Western regions of Ghana, insights from this study could be useful for the design of interventions to address the problem of neonatal sepsis in the locale and similar context.

## Methods

### Study setting and design

The study involved a retrospective cross-sectional review of medical records at neonatal intensive care unit (NICU) of the Cape Coast Teaching Hospital (CCTH) from January 1, 2018 to December 31, 2021. The hospital (CCTH) was established in 1998 as the Central regional hospital and later upgraded to the status of a teaching hospital for the training of various cadres of health professionals including doctors and nurses [16]. The facility is currently the largest referral centre in the central region with a 400-bed capacity and provides a variety of health care services including out-patient care, in-patient care, emergency care as well as specialist clinics.

The NICU from which data for the current study was sourced is housed within the Pediatric care ward for the management and treatment of critical neonatal disorders. The unit has a cot capacity of 20 and is equipped with four (4) incubators and six (6) phototherapy devices. An average of 80 neonates are admitted to the NICU on a monthly basis with neonatal sepsis among the top ten (10) indication for admission in the unit.

### Study population and sampling

The target population for the study was neonates hospitalized at NICU of CCTH from January 1, 2018 to December 31, 2021. Neonatal sepsis was diagnosed based on laboratory investigations and the WHO Integrated Management of Neonatal and Childhood Illness (IMNCI) clinical features. The IMNCI clinical signs for diagnosis of neonatal sepsis include either fever (37.5°C) or hypothermia (35.5°C), tachypnea (60 breaths per minute), poor feeding, severe chest in-drawing, lethargy, convulsion, diminished sucking, and unconsciousness [17]. A sampling frame was constructed from the hospital’s electronic health records (EHR) system which contained data on all neonatal admissions for the period under review obtained from the biostatics unit of the hospital. The sampling frame consisted of 3,455 cases from which a minimum sample size of 360 was estimated using Yemane’s formula;

n = N / 1+N (e)^2^, where: n = sample size; N = the population size; e = the acceptable sampling error *95% confidence level and p = 0.5 are assumed [18].

Accordingly, n = 3,455/1+ 3,455(0.05)^2^ = 358.49 ~ 360.

Using allocations proportional-to-admission per year, the number of cases to be sampled for each respective year under review was determined as presented in Table 1. For instance, 670 neonatal cases admitted in the year 2018, represented about 19.4% of the 3,455 cases from 2018–2021. This translated into 70 cases sampled for the year 2018. The respective number of neonatal cases for each year were then selected based on the folder identification numbers for each neonatal case using the simple random sampling function in STATA 16.0 software.

**Table 1:** Sample distribution.

Year	Admissions	Proportion (%)	Sample allocation
2018	670	19.4	70
2019	1022	29.6	106
2020	1072	31.0	112
2021	691	20.0	72
**Total**	**3,455**	**100**	**360**

### Data extraction and analysis

Following the sampling of neonatal cases from the hospital’s electronic health records (EHR), a data extraction form was designed in Microsoft Excel for the extraction of relevant information for the purpose of the study. The form comprised of two (2) main sections. The first section focused on extracting socio-demographic information of the neonates and their mothers (age of mother, age of neonates, marital status, education, occupation, and residence). This was followed by the section on clinical information relating to the neonate (sex of the new-born, gestation time, Apgar score, neonatal jaundice, birth asphyxia, birthweight, neonatal resuscitation) and their mothers (mode of delivery, premature rupture of membranes (PROM), maternal infection, meconium stained liquor, chorioamnionitis, prolonged labour, foul-smelling liquor, breastfeeding, bubble continuous positive air pressure (B-CPAP), oxygen via mask, oxygen via nasal prongs), including a sub-section on the final clinical diagnosis (neonatal sepsis or otherwise).

Upon extraction, the data was transferred into STATA 16.0 software for analyses. The analyses involved the use of both descriptive and inferential statistical techniques. Descriptive analysis involved the use of frequencies and proportions to describe the various factors (socio-demographic and clinical characteristics of neonates and index mothers) the prevalence of sepsis as well as the proportional distribution of sepsis across the various factors considered in the study. With respect to inferential statistics, multivariable binary logistic regression analyses were conducted to determine the factors associated with neonatal sepsis. Statistical significance was set at P<0.05 with odds ratios used to interpret the associations found between the various factors and neonatal sepsis.

### Ethical consideration

Ethical approval for the study was obtained on 14^th^ February, 2020 from the Institutional Review Board of the CCTH. Further, administrative approvals were granted by the management of CCTH, while biostatistics department ensured that all neonatal records were anonymized before the release of the data on for the purpose of this study.

## Results

### Characteristics of neonates and index mothers

As shown in Table 2, the mean age of neonates was 3.04±4.32 days old, with majority (78%) of them aged 0–7 days old, and more than half of neonates (62.5%) being female. The mean age of the mothers was 27.23±5.92 years, with about half (51.4%) of them aged 20–29 years. More than four-in-ten mothers (43.3%) had completed basic school, about two-thirds (63.9%) were married, 54% were employed, and over six-in-ten (66%) lived in urban areas.

**Table 2:** Characteristics of neonate and their index mothers.

Variables	Frequency (n)	Percent (%)
**Age of neonate**	Mean (3.04±4.32)	
0–7 days	281	78.1
8–28 days	79	21.9
**Sex of neonate**		
Male	135	37.5
Female	225	62.5
**Age of mother**	Mean (27.23±5.92)	
<20 years	79	21.9
21–30	185	51.4
31–40	96	26.7
**Education**		
No education	91	25.3
Basic level (Primary/JHS)	156	43.3
Secondary level	84	23.3
Tertiary	29	8.1
**Marital status**		
Single	130	36.1
Married/Cohabiting	230	63.9
**Occupation**		
Employed	195	54.2
Unemployed	165	45.8
**Residence**		
Urban	236	65.6
Rural	124	34.4
**Gestational age**		
Term	163	45.3
Preterm	197	54.7
**Apgar score**		
Low range (<6)	233	64.7
Normal range (>7)	127	35.3
**Neonatal weight**		
Low birth weight (<2.5)	197	54.7
Normal birth weight (2.5–3.5)	128	35.7
Overweight (>3.5)	35	9.7
**Birth asphyxia**		
Yes	150	41.7
No	210	58.3
**Neonatal jaundice**		
Yes	125	34.7
No	235	65.3
**Neonatal resuscitation**		
Yes	125	34.7
No	235	65.3
**Mode of delivery**		
C/S	104	28.9
SVD	256	71.1
**PROM**		
Yes	96	26.7
No	264	73.3
**Maternal infection**		
Yes	133	36.9
No	227	63.1
**Meconium liquor**		
Yes	91	25.3
No	269	74.7
**Chorioamnionitis**		
Yes	40	11.1
No	320	88.9
**Prolonged labour**		
Yes	152	42.2
No	208	57.8
**Foul-smelling liquor**		
Yes	113	31.4
No	247	68.6
**Early breastfeeding**		
Yes	151	41.9
No	209	58.1
**B-CPAP**		
Yes	74	20.6
No	286	79.4
**Oxygen via nasal prongs**		
Yes	96	26.7
No	264	73.3
**Oxygen via mask**		
Yes	81	22.5
No	279	77.5

With respect to the clinical characteristics, more than half (55%) of neonates were preterm births, approximately 65% had an Apgar score lower than six (6), and 55% of them had a low birth weight (<2.5kg). About four-in-ten (42%) of the neonates had birth asphyxia and 35% of them were jaundiced or resuscitated. Majority (71%) of the index mothers delivered their neonates through spontaneous vaginal delivery (SVD), with over a quarter (27%) of them experiencing PROM and 37% having maternal infection. Again, about 25% had meconium liquor, 11% had chorioamnionitis, 42% had prolonged labour, 31% had history of foul-smelling liquor and about 42% initiated early breastfeeding. About a fifth (20%) of the neonates required bubble continuous positive airway pressure (B-CPAP), and about 27% and 23% receiving oxygen via nasal prongs and mask, respectively.

### Prevalence of neonatal sepsis

**Fig 1:**
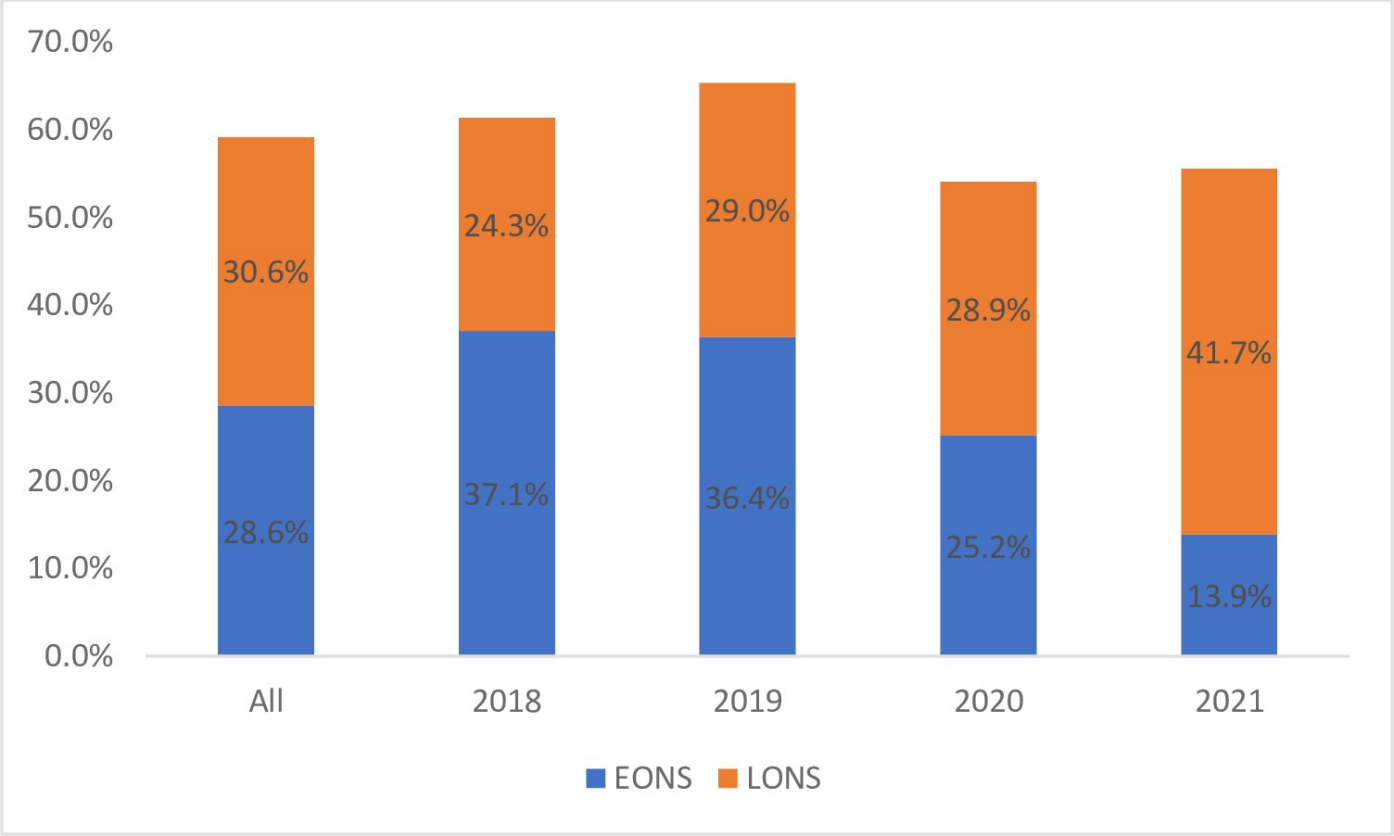
Prevalence of neonatal sepsis from 2018–2021. EONS = Early onset neonatal; LONS = Late onset neonatal sepsis.

The period (2018–2021) prevalence of neonatal sepsis from the sample of 360 was 59%, with early-onset neonatal sepsis (EONS) and late-onset neonatal sepsis (LONS) accounting for about 29% and 30%, respectively. These estimates translate to an average yearly prevalence of neonatal sepsis of 15%. Nonetheless, the disaggregated results as depicted in Fig 1 indicate that the highest prevalence of neonatal sepsis was observed in 2019 (65%). Further, EONS reduced over the period from 37% in 2018 to about 14% in 2021, while LONS increased from 24% to about 42% over the same period.

### Neonatal sepsis by characteristics of neonates and index mothers

Table 3 presents the distribution of neonatal sepsis across the various characteristics of neonates and their index mothers. Neonatal sepsis was higher among neonates older than one week (70%) and among female neonates (60%). On the other hand, neonatal sepsis reduced as age and educational level of mother increased. For instance, neonatal sepsis reduced from 71% among mothers younger than 20 years to about 52% mothers aged 30 years or more; and from 68% among mothers without education to about 38% among those with tertiary level of education. In addition, higher proportion of sepsis was observed among neonates whose mothers were married (61%), employed, (60%) and mothers residing in rural localities (66%). The chi-squared test showed that these observed socio-demographic variations was statistically significant (p<0.05) for age of neonate, age of index mother and mother’s educational level.

**Table 3:** Neonatal sepsis by characteristics of neonates and index mothers.

**Characteristics**	**Neonatal Sepsis (N = 360)**		**Total (N)**	**X** ^**2**^	**P-value**
	**Yes n(%)**	**No n (%)**			
**Age of neonate**					
0–7 days	158(56.2)	123(43.8)	281	4.578	0.032
8–28 days	55(69.6)	24(30.4)	79		
**Sex of neonate**					
Male	78(57.8)	57(42.2)	135	0.172	0.678
Female	135(60.0)	90(40.0)	225		
**Age of mother**					
<20	56(70.9)	23(29.1)	79	6.620	0.037
21–30	107(57.8)	78(42.2)	185		
31–40	50(52.1)	46(47.9)	96		
**Educational level of mother**					
None	62(68.1)	29(31.9)	91	8.509	0.037
Basic level (Primary/JHS)	91(58.3)	65(41.7)	156		
Secondary (SHS/Voc/Tech)	49(58.3)	35(41.7)	84		
Tertiary	11(37.9)	18(62.1)	29		
**Marital status**					
Single	73(56.2%)	57(43.8%)	130	0.764	0.382
Married/Cohabiting	140(60.9%)	90(39.1%)	230		
**Occupation**					
Employed	117(60.0%)	78(40.0%)	195	0.122	0.727
Unemployed	96(58.2%)	69(41.8%)	165		
**Residence**					
Urban	131(55.5%)	105(44.5%)	236	3.795	0.051
Rural	82(66.1%)	42(33.9%)	124		
**Gestational age**					
Term	87(53.4)	76(46.6)	163	4.137	0.042
Preterm	126(64.0)	71(36.0)	197		
**Apgar score**					
Low range (<6)	149(63.9)	84(36.1)	233	6.251	0.012
Normal range (>7)	64(50.4)	63(49.6)	127		
**Neonatal weight**					
Low birth weight (<2.5)	127(64.5)	70(35.5)	197	6.869	0.032
Normal birth weight (2.5–3.5)	71(55.5)	57(44.5)	128		
Overweight (>3.5)	15(42.9)	20(57.1)	35		
**Birth asphyxia**					
Yes	93(62.0)	57(38.0)	150	0.854	0.355
No	120(57.1)	90(42.9)	210		
**Neonatal Jaundice**					
Yes	73(58.4)	52(41.6)	125	0.047	0.829
No	140(59.6)	95(40.4)	235		
**Neonatal resuscitation**					
Yes	69(55.2)	56(44.8)	125	1.247	0.264
No	144(61.3)	91(38.7)	235		
**Mode of delivery**					
C/S	164(64.1)	92(35.9)	256	8.792	0.003
SVD	49(47.1)	55(52.9)	104		
**PROM**					
Yes	54(56.3)	42(43.8)	96	0.461	0.497
No	159(60.2)	105(39.8)	264		
**Maternal infection**					
Yes	89(66.9)	44(33.1)	133	5.245	0.022
No	124(54.6)	103(45.4)	227		
**Meconium liquor**					
Yes	47(51.6)	44(48.4)	91	2.849	0.091
No	166(61.7)	103(38.3)	269		
**Chorioamnionitis**					
Yes	23(57.5)	17(42.5)	40	0.052	0.820
No	190(59.4)	130(40.6)	320		
**Prolonged labour**					
Yes	101(66.4)	51(33.6)	152	5.772	0.016
No	112(53.8)	96(46.2)	208		
**Foul smelling**					
Yes	77(68.1)	36(31.9)	113	5.491	0.019
No	136(55.1)	111(44.9)	247		
**Early breastfeeding**					
Yes	86(57.0)	65(43.0)	151	0.527	0.468
No	127(60.8)	82(39.2)	209		
**B-CPAP**					
Yes	50(67.6)	24(32.2)	74	2.721	0.099
No	163(57.0)	123(43.0)	286		
**Oxygen via nasal prongs**					
Yes	49(52.7)	44(47.3)	93	2.103	0.147
No	163(61.3%)	103(38.7)	266		
**Oxygen via mask**					
Yes	57(70.4)	24(29.6)	81	5.430	0.020
No	156(55.9)	123(44.1)	279		

Chi-square- X^2^; p< 0.05.

With respect to clinical characteristics of neonates, more than six-in-ten hospitalized neonates with each of the following characteristics were diagnosed with neonatal sepsis; late initiation of breastfeeding (61%), low birth weight (65%), low Apgar scores (64%), preterm (64.0%), and asphyxia (62.0%). More than half of the babies with jaundice (58%) and those who were resuscitated after birth (55%) were diagnosed with neonatal sepsis. Regarding maternal clinical characteristics, neonatal sepsis was higher in neonates whose mothers delivered via spontaneous vaginal delivery (SVD) (64%) compared with caesarean section (C/S) (47%). Neonatal sepsis was higher among babies whose mothers had infection (67%), prolonged labour (66%) and foul-smelling liquor (68%), compared with mothers without such clinical characteristics. This was the case for their counterparts whose mothers did not have a history of PROM (60%), meconium liquor (62%), and chorioamnionitis (59%). However, these clinical characteristics were statistically significantly for gestational age, Apgar score, neonatal weight, mode of delivery, maternal infection, prolonged labor, foul smelling and oxygen via mask.

### Factors independently associated with neonatal sepsis

In modeling to determine factors independently associated with neonatal sepsis multivariable logistic analyses, six factors emerged, namely; educational level of mothers, mode of delivery, maternal infection, fouls smelling, neonatal weight and Apgar score (Table 4). Neonates whose index mothers had no formal education (AOR = 2.65; 95% CI:1.04–6.70) had significantly higher odds of developing neonatal sepsis. In addition, the odds of a neonate developing neonatal sepsis were significantly higher for those whose mothers had an infection (AOR = 1.79, 95% CI:1.09–2.94) or foul-smelling liquor (AOR = 1.84, 95% CI:1.09–3.07), with reference to neonates whose mothers had no infection or foul-smelling liquor. On the other hand, neonate who were delivered via caesarian section (C/S) had significantly lower odds of developing neonatal sepsis 0.45 (95% CI:0.26–0.75). Regarding neonatal factors, neonates with a low birth weight (AOR = 2.54; 95% CI: 1.06–6.09) and those with a low Apgar score (AOR = 1.64; 95% CI: 1.01–2.67) had significantly higher odds of developing neonatal sepsis.

**Table 4:** Factors associated with neonatal sepsis.

Variables	COR [95%CI]	P-value	AOR [95%CI]	P-value
**Age of neonate**				
0–7 days	0.56[0.33–0.96]	0.034	0.71[0.39–1.28]	0.252
8–28 days	1			
**Age of mother**				
<20	2.24[1.19–4.20]	0.012	1.82[0.91–3.65]	0.092
21–30	1.26[0.77–2.07]	0.357	1.08[0.62–1.89]	0.796
31–40	1		1	
**Educational level of mother**				
None	3.49[1.47–8.35]	0.005	2.65[1.04–6.70]	0.040
Primary/JHS	2.29[1.01–5.17]	0.046	1.80[0.75–4.32]	0.187
SHS/Voc/Tech	2.29[0.96–5.45]	0.061	1.87[0.75–4.75]	0.189
Tertiary	1		1	
**Mode of delivery**				
C/S	0.50[0.32–0.79]	0.003	0.45[0.26–0.75]	0.003
SVD	1		1	
**Maternal infection**				
Yes	1.68[1.08–2.62]	0.023	1.79[1.09–2.94]	0.020
No	1		1	
**Prolonged labour**				
Yes	1.69[1.10–2.62]	0.017	1.59[0.99–2.57]	0.054
No	1		1	
**Foul smelling**				
Yes	1.75[1.09–2.79]	0.020	1.84[1.09–3.07]	0.020
No	1		1	
**Neonatal weight**				
Low weight	2.42[1.17–5.02]	0.018	2.54[1.06–6.09]	0.037
Normal weight	1.66[0.78–3.53]	0.188	2.09[0.91–4.82]	0.084
Overweight	1		1	
**Apgar score**				
Low range (<6)	1.75[1.13–2.71]	0.013	1.64[1.01–2.67]	0.047
Normal range (>7)	1		1	
**Gestational age**				
Preterm	1.55[1.02–2.37]	0.042	0.86[0.48–1.56]	0.624
Term	1		1	
**Oxygen via mask**				
Yes	1.87[1.09–3.19]	0.021	1.46[0.81–2.64]	0.212
No	1		1	

COR- Crude Odds Ratio; AOR- Adjusted Odds Ratio; p< 0.05

## Discussion

We investigated the prevalence of neonatal sepsis and associated factors using data drawn from the NICU of the Cape Coast Teaching Hospital from 2018 to 2021. We observed that approximately 59% of neonates were diagnosed with sepsis over the period under review. Although varied rates of neonatal sepsis have been reported by different studies across LMICs tend to have higher rates than more advanced countries. Nonetheless, the prevalence in the current study is higher than reports from earlier studies conducted in other LMICs including Haiti (54.8%) [19] and Ethiopia (35%) [20]. The relative variation in the prevalence of neonatal sepsis between our study and other studies could largely be attributable to differences in study design and period of review. For instance, while some studies have applied a case-control design [21] others were based on cohort analysis [22].

Previous studies conducted in Ethiopia [23], Nigeria [24] and in Ghana [21] have reported higher prevalence of EOS compared with LOS, in contrast to our observations in this current study. This points to the possibility differences in source of infections resulting in sepsis. Neonates in prior studies might have been exposed to infections from factors related to hospital environment, while neonates from our study might have been exposed to home/community infections [25, 26]. Indeed, this is evident in the higher LOS observed in our study for the years 2020 and 2021 following the institution of COVID-19 policies requiring the early discharge of women within 24 hours after delivery. Perhaps, this finding highlights the need to emphasize education of women on proper hygiene practices prior to discharge and during postnatal visits.

Although prior extant studies in Ghana [21] and Egypt [27] report no associated between educational level of mothers and neonatal sepsis, our study observed that neonates born to mothers without formal education had a higher propensity of developing neonatal sepsis. Uneducated mothers are less likely to be aware of or adhere to infection control procedures, and may also have less likely to spot danger signs in order to prevent sepsis due [28]. Postnatal counselling and health education on childcare practices tailored to mothers without formal education help in reducing the risk of sepsis among neonates born to such mothers.

As reported in prior studies in India [29] and Ethiopia [30], the current study found a higher probability of neonatal sepsis among neonates with a birth weight below 2.5 kilograms. Neonates with a low birth weight tend to have underdeveloped immune systems, and are therefore susceptible to vertical transmission of organisms from the mother before or during birth, as well as nosocomial infections during the course of hospital care after birth [30]. This is further supported by studies which have found low birth weight infants to have increased gram-negative pathogens and reduced gram-positive pathogen [31, 32]. Our finding linking low Apgar score (<6) to neonatal sepsis resonates with prior studies [21, 33]. It is known that stressful labour conditions reduce the ability of neonates to adopt to extra uterine life, thereby predisposing them to sepsis, especially in the event of immunological insult or revival from asphyxia [34, 35]. This implies that aseptic precautions are strictly adhered to while caring for and performing procedures in infants with low birth weight or low Apgar score.

Similar to the findings of Atlaw et al [36] whiles studying neonatal sepsis in Ethiopia, we found neonates delivered via CS to have lower probability if developing sepsis. Indeed, the literature suggests an indirect pathway between CS delivery and neonatal sepsis, such as through lacerations from sharp instruments during the procedure that may serve portals of entry for microorganisms [37, 38]. The increased risk of neonatal sepsis observed in the current study for neonates whose mothers had a history of maternal infection has similarly been reported in the literature [33, 39]. Such neonates might have acquired sepsis through vertical transmission from their mothers who had an infection during the course of pregnancy. Women typically have a suppressed immune system during pregnancy which exposes them to infections which could be transmitted to the newborn before or during birth if not properly managed.

Neonates who were born to mothers with a history of foul-smelling liquor had greater odds of developing neonatal sepsis, with reference to those born from mothers without a history of foul-smelling liquor. Foul-smelling liquor has similarly been linked with neonatal sepsis in previous studies [40, 41] and suggested to an indication of chorioamnionitis which results in systemic infection when neonates come in contact with it. Early detection and treatment of the mother with chorioamnionitis could reduce the baby’s chances of developing neonatal sepsis.

## Limitation of the study

The study has some inherent limitations as acknowledged in the foregoing. A significant amount of the patient’s history information and biodata were missing. There was also a dearth of laboratory investigation to confirm those who indeed had sepsis. Another limitation was that the information on the LHIMS were entered by different physicians with different years of experience which might lead to information bias.

The findings of the analysis were derived from a tertiary health facility, which limits its representativeness. Thus, more longitudinal research addressing the same contributing factors and management of such illnesses for newborns across all tertiary facilities in Ghana would be more instructive.

## Conclusion

The study highlights a high prevalence of neonatal sepsis over the period with higher rates of late onset sepsis among newborns. A number of factors both at the neonatal (low birth weight and Apgar score) and maternal (caesarean delivery, maternal infection, and foul-smelling liquor) level were found to significantly predict neonatal sepsis. These findings underscore the need for health care providers to improve their routine care and assessment of neonates to curb the incidence of neonatal sepsis. In doing so, particular emphasis should be on neonates with low birth weight and Apgar score and those born to mothers via caesarean section, or mothers with maternal infection, and foul-smelling liquor.
